# Associação do Nível de ST2 Solúvel com Mortalidade em 6 Meses e/ou Hospitalização Recorrente Relacionada a Doenças Cardiovasculares em Embolia Pulmonar

**DOI:** 10.36660/abc.20230040

**Published:** 2024-02-16

**Authors:** Hakan Gunes, Handan Gunes, Musa Dagli, Mehmet Kirişçi, Meryem Özbek, Nurhan Atilla, Mehmet Birhan Yılmaz

**Affiliations:** 1 Kahramanmaras Sutcu Imam University Department of Cardiology Faculty of Medicine Kahramanmaras Turkey Department of Cardiology, Kahramanmaras Sutcu Imam University, Faculty of Medicine, Kahramanmaras – Turkey; 2 Cumnhuriyet University Department of Physiology Faculty of Medicine Sivas Turkey Department of Physiology, Cumnhuriyet University, Faculty of Medicine, Sivas – Turkey; 3 Kahramanmaras Sutcu Imam University Department of Cardiovascular Surgery Faculty of Medicine Kahramanmaras Turkey Department of Cardiovascular Surgery, Kahramanmaras Sutcu Imam University, Faculty of Medicine, Kahramanmaras – Turkey; 4 Kahramanmaras Sutcu Imam University Department of Chest Diseases Faculty of Medicine Kahramanmaras Turkey Department of Chest Diseases, Kahramanmaras Sutcu Imam University, Faculty of Medicine, Kahramanmaras – Turkey; 5 Dokuz Eylul University Department of Cardiology Faculty of Medicine Izmir Turkey Department of Cardiology, Dokuz Eylul University, Faculty of Medicine, Izmir – Turkey

**Keywords:** Embolia Pulmonar, Mortalidade, Hospitalização

## Abstract

**Fundamento:**

A associação de supressão solúvel da tumorigênese-2 (sST2) com prognóstico em embolia pulmonar (EP) é desconhecida.

**Objetivo:**

Este estudo teve como objetivo investigar a relação entre os níveis de sST2 em pacientes com EP aguda e mortalidade em 6 meses e hospitalizações recorrentes.

**Métodos:**

Este estudo prospectivo incluiu 100 pacientes com EP aguda. Os pacientes foram classificados em dois grupos de acordo com a mortalidade em 6 meses e a presença de hospitalizações recorrentes relacionadas a doenças cardiovasculares. Dois grupos foram comparados. Um valor de p de 0,05 foi considerado estatisticamente significativo.

**Resultados:**

Os níveis de ST2 solúvel foram significativamente maiores no grupo com mortalidade e internações recorrentes. (138,6 ng/mL (56,7-236,8) vs. 38 ng/mL (26,3-75,4); p<0,001) O melhor limite de corte para níveis de sST2 na previsão de um desfecho composto de mortalidade em 6 meses e/ou a hospitalização recorrente relacionada a doenças cardiovasculares foi >89,9, com especificidade de 90,6% e sensibilidade de 65,2%, de acordo com a curva ***Receiver Operating Characteristic*** (área sob a curva = 0,798; IC 95%, 0,705–0,891; p <0,0001). Após ajuste para fatores de confusão que foram estatisticamente significativos na análise univariada ou para as variáveis correlacionadas com os níveis de sST2, nível de sST2 (OR = 1,019, IC 95%: 1,009-1,028, p 0,001) e proteína C reativa (PCR). (OR = 1,010, IC 95%: 1,001-1,021, p = 0,046) continuaram a ser preditores significativos de mortalidade em 6 meses e/ou hospitalização recorrente relacionada a doenças cardiovasculares no modelo de regressão logística múltipla através do método ***backward stepwise***.

**Conclusões:**

O nível de ST2 solúvel parece ser um biomarcador para prever mortalidade em 6 meses e/ou hospitalização recorrente relacionada a doenças cardiovasculares em pacientes com EP aguda.

## Introdução

**Figure:**
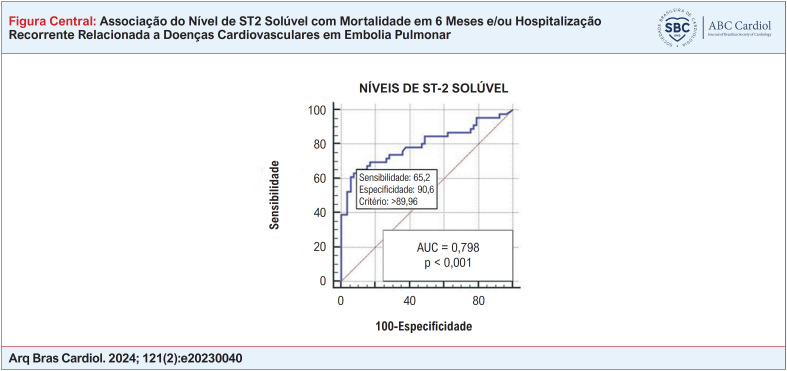


O embolismo pulmonar (EP) é a síndrome cardiovascular mais comum após infarto do miocárdio e acidente vascular cerebral, com incidência anual variando entre 0,1% e 0,2% nos países europeus.^1^ Como os sintomas iniciais da EP são inespecíficos, a taxa de mortalidade varia entre 1% e 60% dependendo da apresentação clínica e das características do paciente.^2,3^ Dados de estudos anteriores revelaram a presença de disfunção ventricular direita e instabilidade hemodinâmica evidente como importante preditor de mortalidade em pacientes com EP aguda.^4,5^ Biomarcadores de instabilidade hemodinâmica evidente e função ventricular direita têm sido de interesse recentemente.^6-8^ Concordantemente, foi demonstrada a relação entre troponina cardíaca, BNP e níveis de FABP do tipo cardíaco com o curso clínico da EP e mortalidade.^9-11^

ST2 é um ligante para IL-33, pois pertence à família de receptores de interleucina (IL) 1. Existem duas isoformas primárias, nomeadamente, solúvel (sST2) e transmembranar ou celular (ST2L). O sistema cardioprotetor, auxiliando na prevenção da hipertrofia e apoptose dos cardiomiócitos, inclui a IL-33 e seu receptor. O ST2 solúvel liga-se à IL-33 e inibe seus efeitos a jusante, levando a um aumento nas concentrações de IL-33 em pacientes com estresse cardiovascular e fibrose.^12–16^ Demonstrou-se que o aumento dos níveis de sST2 está relacionado a maior mortalidade e morbidade em pacientes com doença arterial coronariana (DAC),^17^ aguda^18,19^ e insuficiência cardíaca (IC) crônica,^20,21^ e hipertensão arterial pulmonar.^22^ A relação entre os níveis de sST2 e o curso da doença em pacientes com EP é desconhecida.

Este estudo teve como objetivo investigar a relação entre os níveis de sST2 e a mortalidade em 6 meses e/ou hospitalização recorrente relacionada a doenças cardiovasculares em pacientes com EP aguda.

## Métodos

Este estudo foi concebido como um estudo prospectivo estudo de coorte. No estudo, foram incluídos 100 pacientes que foram internados no pronto-socorro do Hospital Universitário Kahramanmaraş Sutcu İmam entre dezembro de 2018 e outubro de 2019 e com diagnóstico de EP aguda com angiotomografia computadorizada. Os critérios para inclusão no estudo foram ter idade superior a 18 anos e participar do estudo mediante obtenção de consentimento informado por escrito. Foram excluídos do estudo aqueles com história prévia de IC, síndrome coronariana aguda no momento da admissão, insuficiência renal crônica, portadores de hipertensão pulmonar, portadores de doença autoimune congênita, em uso de corticosteroides e com diagnóstico de sepse. Os achados clínicos na admissão, características demográficas, resultados laboratoriais, tratamentos e acompanhamento dos pacientes foram registrados por meio de um questionário padronizado por investigadores cegos aos níveis de biomarcadores. Exames bioquímicos foram realizados no momento da internação. Um aplicativo semelhante foi aplicado para cálculo da pontuação PESI durante a solicitação ao pronto-socorro. Exames eletrocardiográficos e ecocardiográficos dos pacientes foram realizados no momento da admissão. O tempo de internação e a mortalidade hospitalar foram registrados. De acordo com as recomendações das diretrizes, a terapia anticoagulante oral foi iniciada na alta e os pacientes foram acompanhados. Os pacientes que receberam alta foram acompanhados regularmente a cada três meses para monitorar o desenvolvimento de hipertensão pulmonar tromboembólica crônica.

O *endpoint* primário neste estudo foi mortalidade por todas as causas (MTC) em 6 meses e/ou hospitalizações recorrentes relacionadas a doenças cardiovasculares durante o período de acompanhamento de 6 meses. IC, síndrome coronariana aguda, fibrilação ventricular e atrial, embolia pulmonar recorrente, edema pulmonar, hipertensão pulmonar e hipertensão pulmonar tromboembólica crônica foram consideradas internações recorrentes relacionadas a doenças cardiovasculares.

### Ensaios de biomarcadores

As amostras foram coletadas por punção venosa periférica em tubos contendo EDTA, centrifugadas imediatamente e armazenadas a–80°C para análises posteriores. A avaliação dos níveis de sST2 foi realizada em amostras basais usando um imunoensaio monoclonal sanduíche altamente sensível (Aspect-PLUS ST2 Rapid Test, TM) com características de limite inferior de detecção de 12,5 ng/mL, limite superior de detecção de 250 ng/mL, um coeficiente de variação intraensaio de 10,4% e um coeficiente de variação interensaio de 13,6%.

Para medições de D-Dimer, amostras de sangue coletadas em tubos de citrato de sódio foram centrifugadas a 4.000 rpm por 10 minutos e seu plasma foi separado. Os níveis de D-Dimer foram medidos a partir do plasma obtido pelo método imunoturbidimétrico, utilizando um kit Innovance D-Dimer e um analisador de coagulação Sysmex CS 2000i (Sysmex Corporation, Kobe, Japão).

A análise do NT pró-BNP foi avaliada com o aparelho AQT 90 flex (Radiometer Medical Aps, Bronshoj, Dinamarca).

Os níveis de PCR foram determinados quantitativamente pelo método imunonefelométrico utilizando kit apropriado em aparelho Siemens BN II (Alemanha).

Os níveis de troponina foram determinados pelo imunoensaio enzimático de micropartículas de troponina ADV da Abbott no Analisador de Imunoensaio Architect i2000SR (Abbott Diagnostics, Chicago, Illinois, EUA).

A medição da atividade da CK-MB foi realizada pelo método de inibição imunológica utilizando instrumentos Abbott Architect c 8000 (Abbott Diagnostics, Chicago, Illinois, EUA).

As medidas de procalcitonina (PCT) foram medidas a partir do soro pelo método de quimioluminescência usando o dispositivo Siemens Bayer Advia Centaur CP Immunoassay System (Nova York, EUA) e o kit PCT (BRAHMS Diagnostica, Berlim, Alemanha).

### Ecocardiografia

Ecocardiografistas especialistas, cegos ao plano de estudo, realizaram exames de ecocardiografia transtorácica utilizando o sistema de ultrassonografia cardíaca Vivid 7® (GE VingMed Ultrasound AS; Horten, Noruega) com sondas de 2,5 a 5 MHz. A imagem ecocardiográfica foi realizada em posição lateral esquerda e supina com eixos paraesternal longo e curto, e janelas apicais e subcostais foram utilizadas para obtenção de traçados Doppler e imagens 2D. As medidas incluíram fração de ejeção do VE (FEVE; método de Simpson modificado), pressão sistólica da artéria pulmonar (PSAP), excursão sistólica do plano anular tricúspide (TAPSE), diâmetro da artéria pulmonar (DAP) e alteração da área fracionada do ventrículo direito (FAC do VD) conforme diretrizes da Sociedade Americana de Ecocardiografia.^23^

### Análise estatística

Em todas as análises estatísticas foi utilizado o pacote de software SPSS versão 14 (SPSS Inc., Chicago, IL, EUA, institucional). O valor p bilateral < 0,05 foi considerado estatisticamente significativo. Número e porcentagem foram utilizados na expressão das variáveis categóricas; as variáveis contínuas foram apresentadas como média±desvio padrão (DP) ou mediana e intervalo interquartil (IIQ), dependendo da normalidade de sua distribuição. O teste de Kolmogorov-Smirnov foi utilizado para determinar o pressuposto de normalidade dos dados. Na comparação das variáveis contínuas foram utilizados teste t para amostras independentes e teste U de Mann-Whitney conforme normalidade dos dados. Para comparar os dados categóricos, foi utilizado um teste qui-quadrado apropriado. Nas análises de correlação, preferiu-se o teste de correlação de Pearson para variáveis com distribuição normal e o teste de correlação de Spearman para variáveis com distribuição não normal. Para a previsão de mortalidade em 6 meses e/ou hospitalização recorrente relacionada a doenças cardiovasculares usando a análise da curva receiver-operator-characteristic (ROC) MedCalc (v12.7.8, registro pessoal), foi encontrado um limite de corte ideal para o nível de sST2, que foi alcançado através da determinação da área sob a curva (AUC) com um intervalo de confiança de 95%. O melhor valor de corte para mortalidade em 6 meses e/ou internação recorrente por doenças cardiovasculares foi obtido pelo cálculo da maior soma de sensibilidade e especificidade-1. Utilizando o método *enter*, foi construído um modelo de regressão logística com uma única variável. Utilizando o teste de Pearson para dados paramétricos e o teste de Spearman para dados ordinais, foi determinada correlação univariada. Uma análise univariada foi realizada para o desfecho primário neste estudo. Para determinar os preditores independentes de mortalidade em 6 meses e/ou hospitalização recorrente por doença cardiovascular, as variáveis que tiveram associação significativa na análise univariada foram inseridas no modelo de regressão logística multivariada usando o método *backward stepwise* juntamente com outros potenciais confundidores.

## Resultados

A idade média dos 100 pacientes (39 homens, 61 mulheres) incluídos no estudo foi de 65,5±17,5 anos. Um total de 27 (27%) pacientes morreram, 6 deles (6%) durante a internação índice e 21 (21%) morreram durante o acompanhamento de 6 meses. Dezenove pacientes tiveram internações. Os pacientes foram classificados em dois grupos: grupo 1, incluindo pacientes (46) com desfecho composto de mortalidade em 6 meses e/ou hospitalização recorrente relacionada a doenças cardiovasculares durante o acompanhamento, e pacientes do grupo 2 (54) sem desfecho composto. No primeiro grupo, 26 pacientes foram incluídos devido à mortalidade em 6 meses e 20 pacientes devido a hospitalizações relacionadas a doenças cardiovasculares. Os dois grupos foram semelhantes em termos de idade, índice de massa corporal (IMC) durante a internação índice, distribuição por sexo, hipertensão (HT), diabetes mellitus (DM), tabagismo, presença de câncer e trombose venosa profunda. A síncope na admissão foi significativamente mais frequente no grupo 1. Os escores PESI dos pacientes do grupo 1 foram estatisticamente significativamente maiores do que os do grupo 2. (Tabela 1) 7% dos pacientes foram classificados como de muito baixo risco, 9% como tendo um risco baixo, 11% como tendo um risco médio, 14% como tendo um risco elevado e 59% como tendo um risco muito elevado. A análise da distribuição entre os grupos revelou que 37 pacientes do grupo com mortalidade e readmissão apresentavam risco muito alto, 4 apresentavam risco alto, 1 apresentava risco médio, 3 apresentavam risco baixo e 1 apresentava risco muito baixo. No grupo sem mortalidade e readmissão, seis pacientes apresentaram risco muito baixo, seis tiveram risco baixo, dez tiveram risco intermediário, dez tiveram risco alto e doze tiveram risco alto. No grupo de pacientes com readmissão e mortalidade, 35 dos 41 pacientes de alto e muito alto risco receberam terapia trombolítica. 17 dos 22 pacientes de muito alto e alto risco do grupo receberam terapia trombolítica sem hospitalização recorrente ou mortalidade. Em ambas as categorias, todos os pacientes com risco extremamente elevado receberam terapia trombolítica e não houve mortes devido a complicações relacionadas à terapia trombolítica. Seis pacientes desenvolveram hipertensão pulmonar tromboembólica crônica durante o acompanhamento de seis meses. Após avaliação dos níveis de sST2, o grupo que desenvolveu HPTEC apresentou níveis de sST2 significativamente mais elevados. (172,7±75,6 vs 88,8±75,9 ng/mL; p= 0,041). Em nosso estudo, a taxa de mortalidade hospitalar foi de 12%. A taxa de mortalidade no seguimento de 6 meses foi de 26%.

**Tabela 1:** Características basais, significância laboratorial, parâmetros ecocardiográficos

Características		Mortalidade em 6 meses e/ou hospitalização cardiovascular recorrente (n:46)	Nenhuma mortalidade em 6 meses e/ou hospitalização cardiovascular recorrente (n:54)	P
Anos de idade		68,9±15,7	62,6±18,6	0,076
Sexo		18 (%39)	21 (%40)	1.000
IMC, kg/m^2^		27,6±4,0	28,3±3.2	0,743
Hipertensão		23 (%50)	24 (%45)	0,639
Diabetes mellitus		12 (%26)	7 (%13)	0,172
Hiperlipidemia		17 (%37)	13 (%25)	0,267
Fumante		11 (%24)	10 (%19)	0,741
Câncer		14 (%30)	7 (%13)	0,065
TVP		18 (%39)	28 (%53)	0,173
Síncope		15 (%33)	6 (%11)	0,019
Dor no peito		20 (%44)	16 (%30)	0,245
S1Q3T3		6 (%13)	9 (%17)	0,792
Escore PESI		181±71	127±59	<0,001
**Significado laboratorial**				
	PCR, mg/L	73 (21,6-134)	41 (16,8-88)	0,040
	RPC, ng/mL	0,11 (0,05-0,55)	0,07 (0,02-0,14)	0,012
	NT-pro BNP, pg/mL	992 (397-2855)	360 (233-1495)	0,009
	Dímero D, ng/mL	4,7 (2,0-12,6)	4,1 (1,7-11,6)	0,664
	CK-MB, ng/mL	3,5 (1,5-5,8)	1,7 (0,9-4,3)	0,040
	Troponina I, ng/mL	0,09 (0,01-0,95)	0,04 (0,02-0,47)	0,624
	sST-2,ng/ml	138,6 (56,7-236,8)	38 (26,3-75,4)	<0,001
**Parâmetros ecocardiográficos**				
	TAPSE, mm	13.3±3.5	16±3.8	0,001
	sPAB, mmHg	40±9,9	37±9,9	0,150
	FE VR, %	36,6±7,5	38,6±7.7	0,206
	Diâmetro AP, mm	29.2±4.6	27.2±4.2	0,026
	Diâmetro VD, mm	38,2±6.3	35.1±6.4	0,001

IMC: índice de massa corporal; TVP: trombose venosa profunda; S1Q3T3: D1 onda S, D3 onda Q, D3 onda T; PESI: índice de gravidade de embolia pulmonar; PCR: proteína C reativa; PRC: procalcitonina; NT-pro BNP: n -peptídeo natriurético cerebral pró-hormonal terminal; CK-MB: creatina quinase–MB; TAPSE: excursão sistólica do plano anular tricúspide; sPAB: pressão sistólica da artéria pulmonar; FE: fração de ejeção; VD: ventrículo direito; AP: artéria pulmonar.

Nos exames laboratoriais, os níveis de PCR, procalcitonina, pró-BNP, CK-MB e sST2 foram maiores no grupo 1 em comparação ao grupo 2, respectivamente (Tabela 1).

Na avaliação ecocardiográfica, a fração de ejeção (FE) do ventrículo direito, a pressão sistólica da artéria pulmonar e a FE do ventrículo esquerdo foram semelhantes em ambos os grupos. O TAPSE foi significativamente menor no grupo 1 do que no grupo 2. O diâmetro do ventrículo direito e o diâmetro da artéria pulmonar foram significativamente maiores no grupo 1 do que no grupo 2, respectivamente (Tabela 1).

Embora os níveis de sST2 tenham se correlacionado positivamente com idade, escore PESI, pressão sistólica da artéria pulmonar, diâmetro da artéria pulmonar, troponina e CK-MB, foram negativamente correlacionados com o TAPSE (Tabela 2).

**Tabela 2:** Coeficientes de correlação para níveis solúveis de ST-2

**Idade**	0,212	0,035
**PESI**	0,459	<0,001
**TAPSE**	-0,408	<0,001
**sPAB**	0,280	0,005
**Diâmetro AP**	0,258	0,010
**CK-MB**	0,316	0,001
**Troponina I**	0,276	0,006

PESI: índice de gravidade da embolia pulmonar; TAPSE: excursão sistólica do plano do anel tricúspide; sPAB: pressão sistólica da artéria pulmonar; AP: artéria pulmonar; CK-MB: creatina quinase–MB.

O melhor limite de corte para os níveis de sST2 na predição de mortalidade em 6 meses e/ou hospitalizações recorrentes relacionadas a doenças cardiovasculares foi >89,9 ng/mL com especificidade de 90,6% e sensibilidade de 65,2%, de acordo com o curva característica de operação do receptor (área sob a curva = 0,798; IC 95%, 0,705–0,891; p <0,0001). (Figura Central)

**Figura 1:**
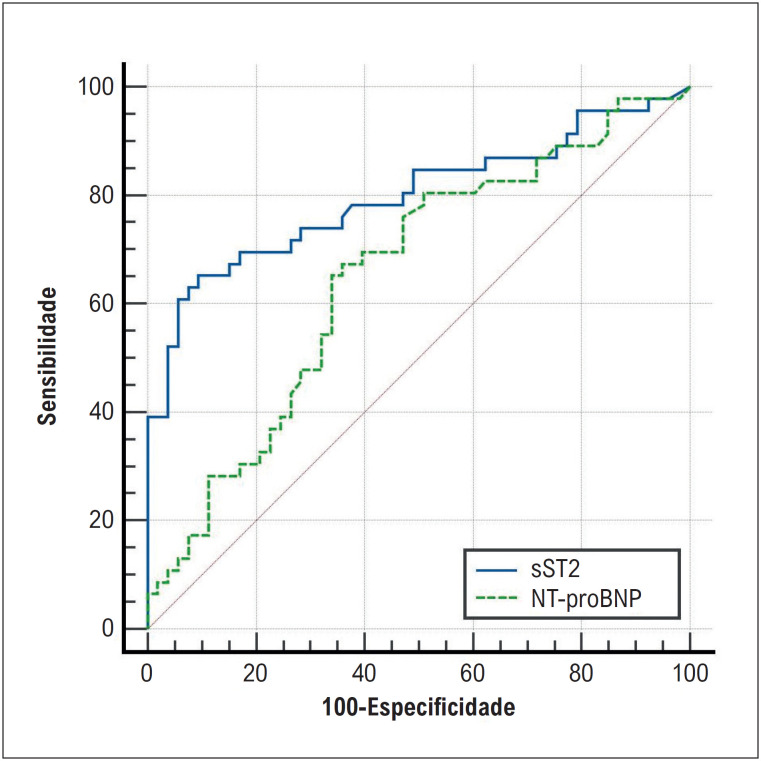
Curva característica do operador do receptor (ROC) dos níveis de ST-2 solúvel e níveis de NT-pro BNP para prever mortalidade em 6 meses e/ou hospitalização cardiovascular recorrente.

A Figura 1 também mostra a comparação entre pró-BNP, marcador direto de sobrecarga ventricular, e sST2 (p=0,009).

**Figura 2:**
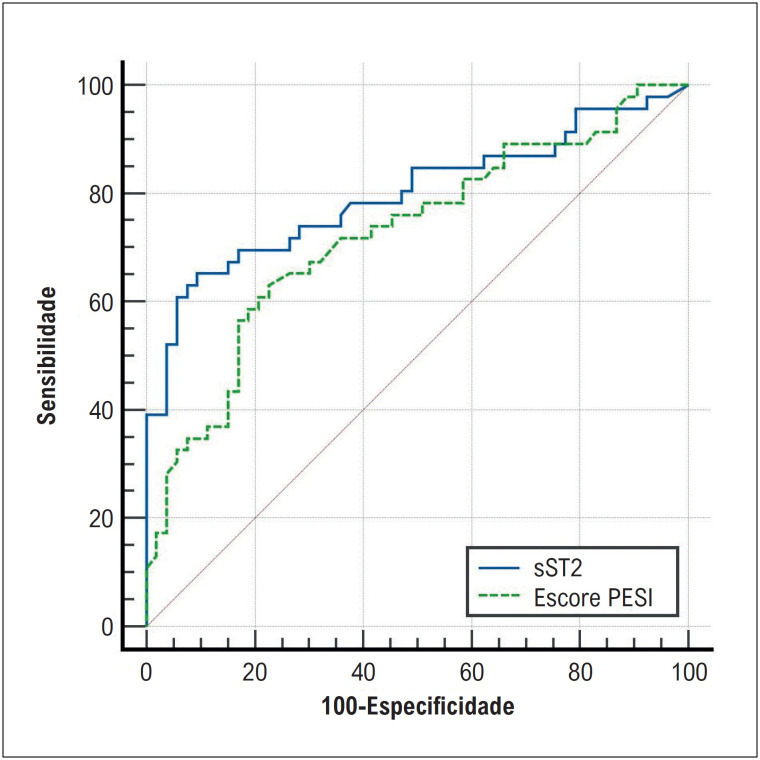
Curva característica do operador do receptor (ROC) dos níveis de ST-2 solúvel e pontuação PESI para prever mortalidade em 6 meses e/ou hospitalização cardiovascular recorrente.

A Figura 2 mostra a comparação entre o PESI, que determina o risco de mortalidade e a gravidade das complicações e sST2 (p=0,175).

Após ajuste para fatores de confusão que foram estatisticamente significativos na análise univariada ou para as variáveis correlacionadas com os níveis de sST2, o nível de sST2 e a PCR continuaram a ser preditores significativos de um desfecho composto de mortalidade em 6 meses e/ou doença cardiovascular recorrente. internações no modelo de regressão logística múltipla via método *backward stepwise* (Tabela 3).

**Tabela 3:** Análise de regressão logística univariada e multivariada representando os preditores independentes de mortalidade em 6 meses e/ou internação cardiovascular recorrente

Variáveis		Univariada		Multivariada	
		OR (IC 95%)	p	OR (IC 95%)	p
sST-2		1,021 (1,012-1,030)	<0,001	1,019 (1,009-1,028)	<0,001
PCR		1,009 (1,002-1,017)	0,014	1,010 (1,001-1,021)	0,046
NT-proBNP,pg/ml		2,838 (1,281-6,286)	0,010		
Procalcitonina		1,008 (0,914-1,111)	0,878		
CK-MB		1,140 (0,993-1,309)	0,064		
PESI		1,012 (1,006-1,011)	<0,001		
Síncope		3,790 (1,327-10,829)	0,013		
TOQUE		0,831 (0,744-0,928)	0,001		
Diâmetro PA, mm		1,108 (1,010-1,215)	0,029		
Diâmetro VD, mm		1,079 (1,011-1,152)	0,021		
**Valor correlacionado com sST2**					
	Anos de idade	1,022 (0,997-1,046)	0,080		
	TROPONINA I,ng/ml	1,216 (0,786-1,881)	0,380		

PCR: proteína C reativa; NT-PRO BNP: peptídeo natriurético cerebral pró-hormônio n-terminal; CK-MB: creatina quinase–MB; PESI: índice de gravidade da embolia pulmonar; TAPSE: excursão sistólica do plano anular tricúspide.

## Discussão

Neste estudo, o nível de sST2 foi um preditor independente do desfecho composto de mortalidade em 6 meses e/ou hospitalização recorrente por causa cardiovascular em pacientes com EP aguda. Além disso, os níveis séricos de PCR também foram considerados um preditor independente do resultado composto.

A IL-33 é secretada pelas células após dano e necrose celular, células sob estresse mecânico e no processo inflamatório. A IL-33 liga-se ao ST2L, produzindo transcrição de genes inflamatórios e criando uma resposta inflamatória com a liberação de quimiocinas e citocinas inflamatórias. A resposta inflamatória resulta em apoptose e fibrose. Já o ST2 solúvel liga-se à IL-33 e suprime essa resposta inflamatória, gerando assim um mecanismo de defesa para suprimir a apoptose e a fibrose.^24-28^ A principal fonte de sST2 circulante em indivíduos saudáveis e pacientes com diversas doenças não está totalmente elucidada. Dados experimentais sugeriram que o estresse mecânico poderia induzir a expressão de IL-33 e sST2 em cardiomiócitos e fibroblastos.^29^ Foi demonstrado que os níveis de sST2 estavam ligados à mortalidade e ao prognóstico na IC aguda e crônica em relação ao estresse mecânico e aos processos inflamatórios dentro do ventrículo esquerdo.^19-21,30^ Além disso, os níveis de sST2 em pacientes com HP demonstraram estar intimamente relacionados aos parâmetros hemodinâmicos, especialmente à disfunção ventricular direita, e ser um marcador de mortalidade.^22^ Da mesma forma, em nosso estudo, os níveis de sST2 foram considerados um preditor independente de desfechos compostos em Pacientes com EP. A obstrução por trombo na EP gera espaços mortos no pulmão, provoca processo inflamatório e hipóxia. O processo inflamatório e a hipóxia causam vasoconstrição no leito pulmonar, resultando em aumento da resistência vascular pulmonar (RVP) e da pressão arterial pulmonar, o que pode aumentar a pressão de enchimento no ventrículo direito e, consequentemente, aumentar os níveis de sST2. Portanto, o sST2 pode ser um bom biomarcador para refletir a carga ventricular direita. É digno de nota que a carga ventricular direita é um marcador de mortalidade em pacientes com EP.^4-6^ Portanto, os níveis de sST2 podem ser usados como substitutos para resultados insatisfatórios. Pelo contrário, durante o processo inflamatório relacionado à EP aguda, o sST2 pode ligar-se à IL-33 e desempenhar um papel na supressão da inflamação, fibrose e prevenção da remodelação. Houve evidências de apoio em relação ao sST2 derivado do endotélio vascular na literatura.^31^

Há muito se reconhece que a inflamação desempenha um papel na patogênese da trombose arterial e venosa, ativando cascatas de coagulação através da geração de trombina e deposição de fibrina. A trombose arterial e venosa é influenciada por mediadores inflamatórios, especificamente PCR, IL-6, IL-8 e fator de necrose tumoral.^32,33^ Em investigações limitadas em animais, foi demonstrado que a inflamação após EP contribui para lesão, disfunção do VD e inflamação cardíaca.^34^ A embolia pulmonar pode induzir reações inflamatórias vasculares via tromboembolismo da artéria pulmonar, bem como inflamação do parênquima pulmonar via infarto pulmonar mimetizando pneumonia. O tromboembolismo da artéria pulmonar pode acelerar a disfunção do VD através da inflamação cardíaca que contribui para a lesão dos miócitos. A proteína C reativa pode ser usada para avaliar o risco e o prognóstico de isquemia miocárdica e acidente vascular cerebral isquêmico com base na fisiopatologia da trombose arterial.^33^ Também foi demonstrado em estudos anteriores que a PCR elevada, outro marcador inflamatório, também está associada a um mau prognóstico em pacientes com EP.^32,33^ Em nosso estudo, os níveis de PCR foram associados à mortalidade em 6 meses e/ou hospitalizações recorrentes por doenças cardiovasculares.

Pro-BNP, que aumenta em resposta à tensão ventricular direita; CK-MB, que indica lesão do ventrículo direito; e a procalcitonina, que aumenta como resultado do processo inflamatório na embolia pulmonar, variaram entre os grupos. Da mesma forma, descobriu-se que dados ecocardiográficos como TAPSE, diâmetro da artéria pulmonar e diâmetro do VD, que são indicadores da função ecocardiográfica do ventrículo direito, variam entre os grupos. Os dados ecocardiográficos e laboratoriais foram considerados preditores de mortalidade e readmissões na análise univariada. Diferentemente da literatura, os valores de troponina foram semelhantes nos dois grupos da população do nosso estudo. Como a maioria dos pacientes incluídos no estudo eram pacientes de alto risco, os valores de troponina estavam acima da normalidade, mas foram semelhantes entre os grupos. Apenas sST2 e CRP foram considerados preditores independentes na análise multivariada. Isso pode ocorrer porque nossa instalação é um centro terciário e a maioria de nossos pacientes são pacientes de alto risco. Além disso, tanto a mortalidade como a mortalidade associada a hospitalizações recorrentes podem ter contribuído para a independência do sST2 e da PCR como preditores.

### Limitações

O presente estudo tem algumas limitações. Uma das limitações do nosso estudo é a sua natureza unicêntrica, o que impede que os achados sejam generalizados para a população geral de pacientes com EP aguda com graus variados de gravidade. Além disso, o tamanho da amostra não é grande o suficiente para tirar conclusões definitivas. É digno de nota que outros marcadores inflamatórios, como IL-1, IL-6 e TNF-alfa, que poderiam aumentar o sST2, não foram medidos.

## Conclusão

O nível de sST2 parece um biomarcador que pode ser usado para prever um resultado composto de 6 meses em pacientes com EP aguda de alto risco, confirmando sua estreita relação com a carga ventricular direita e seu papel no processo inflamatório.

## References

[1] Mazzolai L, Aboyans V, Ageno W, Agnelli G, Alatri A, Bauersachs R (2018). Diagnosis and Management of Acute Deep Vein Thrombosis: A Joint Consensus Document from the European Society of Cardiology Working Groups of Aorta and Peripheral Vascular Diseases and Pulmonary Circulation and Right Ventricular Function. Eur Heart J.

[2] Agnelli G, Becattini C. (2010). Acute Pulmonary Embolism. N Engl J Med.

[3] Carson JL, Kelley MA, Duff A, Weg JG, Fulkerson WJ, Palevsky HI (1992). The Clinical Course of Pulmonary Embolism. N Engl J Med.

[4] McIntyre KM, Sasahara AA (1974). Determinants of Right Ventricular Function and Hemodynamics After Pulmonary Embolism. Chest.

[5] Konstantinides S. (2005). Pulmonary Embolism: Impact of Right Ventricular Dysfunction. Curr Opin Cardiol.

[6] Kucher N, Rossi E, Rosa M, Goldhaber SZ (2005). Prognostic Role of Echocardiography Among Patients with Acute Pulmonary Embolism and a Systolic Arterial Pressure of 90 Mm Hg or Higher. Arch Intern Med.

[7] van der Meer RW, Pattynama PM, van Strijen MJ, van den Berg-Huijsmans AA, Hartmann IJ, Putter H (2005). Right Ventricular Dysfunction and Pulmonary Obstruction Index at Helical CT: Prediction of Clinical Outcome During 3-Month Follow-Up in Patients with Acute Pulmonary Embolism. Radiology.

[8] Mansencal N, Joseph T, Vieillard-Baron A, Langlois S, El Hajjam M, Qanadli SD (2005). Diagnosis of Right Ventricular Dysfunction in Acute Pulmonary Embolism Using Helical Computed Tomography. Am J Cardiol.

[9] Binder L, Pieske B, Olschewski M, Geibel A, Klostermann B, Reiner C (2005). N-Terminal Pro-Brain Natriuretic Peptide or Troponin Testing Followed by Echocardiography for Risk Stratification of Acute Pulmonary Embolism. Circulation.

[10] Müller-Bardorff M, Weidtmann B, Giannitsis E, Kurowski V, Katus HA (2002). Release Kinetics of Cardiac Troponin T in Survivors of Confirmed Severe Pulmonary Embolism. Clin Chem.

[11] Puls M, Dellas C, Lankeit M, Olschewski M, Binder L, Geibel A (2007). Heart-Type Fatty Acid-Binding Protein Permits Early Risk Stratification of Pulmonary Embolism. Eur Heart J.

[12] Liew FY, Pitman NI, McInnes IB. (2010). Disease-Associated Functions of IL-33: The New Kid in the IL-1 Family. Nat Rev Immunol.

[13] Villarreal DO, Weiner DB (2014). Interleukin 33: A Switch-Hitting Cytokine. Curr Opin Immunol.

[14] Mueller T, Jaffe AS (2015). Soluble ST2--Analytical Considerations. Am J Cardiol.

[15] Dieplinger B, Mueller T. (2015). Soluble ST2 in Heart Failure. Clin Chim Acta.

[16] Seki K, Sanada S, Kudinova AY, Steinhauser ML, Handa V, Gannon J (2009). Interleukin-33 Prevents Apoptosis and Improves Survival After Experimental Myocardial Infarction Through ST2 Signaling. Circ Heart Fail.

[17] Shimpo M, Morrow DA, Weinberg EO, Sabatine MS, Murphy SA, Antman EM (2004). Serum Levels of the Interleukin-1 Receptor Family Member ST2 Predict Mortality and Clinical Outcome in Acute Myocardial Infarction. Circulation.

[18] Mueller T, Dieplinger B, Gegenhuber A, Poelz W, Pacher R, Haltmayer M. (2008). Increased Plasma Concentrations of Soluble ST2 are Predictive for 1-Year Mortality in Patients with Acute Destabilized Heart Failure. Clin Chem.

[19] Rehman SU, Mueller T, Januzzi JL (2008). Characteristics of the Novel Interleukin Family Biomarker ST2 in Patients with Acute Heart Failure. J Am Coll Cardiol.

[20] Ky B, French B, McCloskey K, Rame JE, McIntosh E, Shahi P (2011). High-Sensitivity ST2 for Prediction of Adverse Outcomes in Chronic Heart Failure. Circ Heart Fail.

[21] Felker GM, Fiuzat M, Thompson V, Shaw LK, Neely ML, Adams KF (2013). Soluble ST2 in Ambulatory Patients with Heart Failure: Association with Functional Capacity and Long-Term Outcomes. Circ Heart Fail.

[22] Zheng YG, Yang T, He JG, Chen G, Liu ZH, Xiong CM (2014). Plasma Soluble ST2 Levels Correlate with Disease Severity and Predict Clinical Worsening in Patients with Pulmonary Arterial Hypertension. Clin Cardiol.

[23] Lang RM, Bierig M, Devereux RB, Flachskampf FA, Foster E, Pellikka PA (2005). Recommendations for Chamber Quantification: a Report from the American Society of Echocardiography`s Guidelines and Standards Committee and the Chamber Quantification Writing Group, Developed in Conjunction with the European Association of Echocardiography, a Branch of the European Society of Cardiology. J Am Soc Echocardiogr.

[24] Weinberg EO (2009). ST2 Protein in Heart Disease: from Discovery to Mechanisms and Prognostic Value. Biomark Med.

[25] Liew FY, Pitman NI, McInnes IB. (2010). Disease-Associated Functions of IL-33: the New Kid in the IL-1 Family. Nat Rev Immunol.

[26] Miller AM, Liew FY (2011). The IL-33/ST2 Pathway--a New Therapeutic Target in Cardiovascular Disease. Pharmacol Ther.

[27] Mueller T, Dieplinger B. (2013). The Presage(®) ST2 Assay: Analytical Considerations and Clinical Applications for a High-Sensitivity Assay for Measurement of Soluble ST2. Expert Rev Mol Diagn.

[28] Villarreal DO, Weiner DB (2014). Interleukin 33: A Switch-Hitting Cytokine. Curr Opin Immunol.

[29] Sanada S, Hakuno D, Higgins LJ, Schreiter ER, McKenzie AN, Lee RT (2007). IL-33 and ST2 Comprise a Critical Biomechanically Induced and Cardioprotective Signaling System. J Clin Invest.

[30] Mueller T, Dieplinger B, Gegenhuber A, Poelz W, Pacher R, Haltmayer M. (2008). Increased Plasma Concentrations of Soluble ST2 are Predictive for 1-Year Mortality in Patients with Acute Destabilized Heart Failure. Clin Chem.

[31] Demyanets S, Kaun C, Pentz R, Krychtiuk KA, Rauscher S, Pfaffenberger S (2013). Components of the Interleukin-33/ST2 System are Differentially Expressed and Regulated in Human Cardiac Cells and in Cells of the Cardiac Vasculature. J Mol Cell Cardiol.

[32] Araz O, Yilmazel Ucar E, Yalcin A, Kelercioglu N, Meral M, Gorguner AM (2016). Predictive Value of Serum Hs-CRP Levels for Outcomes of Pulmonary Embolism. Clin Respir J.

[33] Abul Y, Karakurt S, Ozben B, Toprak A, Celikel T. (2011). C-Reactive Protein in Acute Pulmonary Embolism. J Investig Med.

[34] Wu D, Chen Y, Wang W, Li H, Yang M, Ding H (2020). The Role of Inflammation in a Rat Model of Chronic Thromboembolic Pulmonary Hypertension Induced by Carrageenan. Ann Transl Med.

