# Supplementary material for: Associação do Nível de ST2 Solúvel com Mortalidade em 6 Meses e/ou Hospitalização Recorrente Relacionada a Doenças Cardiovasculares em Embolia Pulmonar
Source: Arq Bras Cardiol. 2024 Feb 16;121(2):e20230040. [Article in Portuguese] doi: 10.36660/abc.20230040 (PMC11081135; doi:10.36660/abc.20230040)
Supplement: Translation

# Translation: Association of soluble ST2 Level with 6-month Mortality and/or Recurrent Cardiovascular-Related Hospitalization in Pulmonary Embolism

**Authors:** Hakan Gunes, Handan Gunes, Musa Dagli, Mehmet Kirişçi, Meryem Özbek, Nurhan Atilla, Mehmet Birhan Yılmaz

**Affiliations:** 1 Kahramanmaras Turkey Department of Cardiology, Kahramanmaras Sutcu Imam University, Faculty of Medicine, Kahramanmaras – Turkey; 2 Sivas Turkey Department of Physiology, Cumnhuriyet University, Faculty of Medicine, Sivas – Turkey; 3 Kahramanmaras Turkey Department of Cardiovascular Surgery, Kahramanmaras Sutcu Imam University, Faculty of Medicine, Kahramanmaras – Turkey; 4 Kahramanmaras Turkey Department of Chest Diseases, Kahramanmaras Sutcu Imam University, Faculty of Medicine, Kahramanmaras – Turkey; 5 Izmir Turkey Department of Cardiology, Dokuz Eylul University, Faculty of Medicine, Izmir – Turkey

**Keywords:** Pulmonary Embolism, Mortality, Hospitalization

## Abstract

**Background:**

The association of soluble suppression of tumorigenesis-2 (sST2) levels with prognosis in pulmonary embolism (PE) is unknown.

**Objective:**

This study aimed to investigate the relationship between sST2 levels in patients with acute PE and 6-month mortality and recurrent hospitalizations.

**Methods:**

This prospective study included 100 patients with acute PE. Patients were classified into two groups according to 6-month mortality and the presence of recurrent Cardiovascular-Related hospitalizations. Two groups were compared. A p-value of 0.05 was considered statistically significant.

**Results:**

Soluble ST2 levels were significantly higher in the group with mortality and recurrent hospitalizations. (138.6 ng/mL (56.7-236.8) vs. 38 ng/mL (26.3-75.4); p<0.001) The best cut-off threshold for sST2 levels in the prediction of a composite outcome of 6-month mortality and/or recurrent Cardiovascular-Related hospitalization was found to be >89.9 with a specificity of 90.6% and a sensitivity of 65.2%, according to the receiver operating characteristic curve (area under the curve = 0.798; 95% CI, 0.705–0.891; p <0.0001). After adjusting for confounding factors that were either statistically significant in the univariate analysis or for the variables correlated with the sST2 levels, sST2 level (OR = 1.019, 95% CI: 1.009-1.028, p 0.001) and C-reactive protein (CRP ) (OR = 1.010, 95% CI: 1.001-1.021, p = 0.046) continued to be significant predictors of 6-month mortality and/or recurrent Cardiovascular-Related hospitalization in the multiple logistic regression model via backward stepwise method.

**Conclusion:**

Soluble ST2 level seems to be a biomarker to predict 6-month mortality and/or recurrent Cardiovascular-Related hospitalization in patients with acute PE.

## Introduction

Pulmonary embolism (PE) is the most common cardiovascular syndrome after myocardial infarction and stroke, with an annual incidence varying between 0.1% and 0.2% in European countries.^1^ Since initial symptoms of PE are non-specific, the mortality rate varies between 1% and 60% depending on the clinical presentation and patient characteristics.^2,3^ Data from previous studies revealed the presence of right ventricular dysfunction and overt hemodynamic instability as an important predictor of mortality in patients with acute PE.^4,5^ Biomarkers of overt hemodynamic instability and right ventricular function have been of interest recently.^6-8^ Concordantly, the relationship between cardiac troponin, BNP, and heart-type FABP levels with the clinical course of PE and mortality has been demonstrated.^9-11^

ST2 is a ligand for IL-33 as it is from the interleukin (IL) 1 receptor family. Two primary isoforms, namely, soluble (sST2) and transmembrane or cellular (ST2L), exist. The cardioprotective system, aiding in the prevention of cardiomyocyte hypertrophy and apoptosis of cardiomyocytes, includes IL-33 and its receptor. Soluble ST2 binds IL-33 and inhibits its downstream effects, leading to an increase in the concentrations of IL-33 in patients with cardiovascular stress, and fibrosis.^12–16^ Increased sST2 levels were shown to be related to higher mortality and morbidity in patients with coronary artery disease (CAD),^17^ acute^18,19^ and chronic heart failure (HF),^20,21^ and pulmonary arterial hypertension.^22^ The relationship between sST2 levels and the course of the disease in PE patients is unknown.

This study aimed to investigate the relationship between sST2 levels and 6-month mortality and/or recurrent Cardiovascular-Related hospitalization in patients with acute PE.

## Methods

This study was designed as a prospective cohort study. In the study, 100 patients who were admitted to the emergency department of Kahramanmaraş Sutcu İmam University Hospital between December 2018 and October 2019 and diagnosed to have acute PE with CT angiography were included. The criteria for inclusion in the study were to be over 18 years old and to participate in the study by obtaining written informed consent. Those with a previous history of HF, acute coronary syndrome at the time of admission, chronic renal failure, those with pulmonary hypertension, those with congenital autoimmune disease, those on corticosteroids, and those diagnosed with sepsis were excluded from the study. The clinical findings at admission, demographic characteristics, laboratory results, treatments, and follow-ups of the patients were recorded using a standardized questionnaire by investigators blinded to biomarker levels. Biochemical tests were performed at the time of admission. A similar application was applied for PESI score calculation during the application to the emergency department. ECG and echocardiographic examinations of the patients were performed at the time of admission. The length of hospital stay and in-hospital mortality were recorded. In accordance with guideline recommendations, oral anticoagulant therapy was initiated at discharge, and patients were followed up. Discharged patients were followed up regularly every three months to monitor the development of chronic thromboembolic pulmonary hypertension.

The primary endpoint in this study was 6-month all-cause mortality (ACM) and/or recurrent cardiovascular-related hospitalizations during the 6-month follow-up period. HF, acute coronary syndrome, ventricular and atrial fibrillation, recurrent PE, lung edema, pulmonary hypertension, and chronic thromboembolic pulmonary hypertension were considered recurrent cardiovascular-related hospitalizations.

### Biomarker assays

Samples were collected via peripheral venepuncture into EDTA-containing tubes, centrifuged immediately, and stored at–80°C for subsequent analyses. The sST2 levels’ evaluation was performed on baseline samples using a highly sensitive sandwich monoclonal immunoassay (Aspect-PLUS ST2 Rapid Test, TM) with features of a 12.5 ng/mL lower limit of detection, a 250 ng/mL upper limit of detection, an intra-assay coefficient of variation of 10.4% and an inter-assay coefficient of variation of 13.6%.

For D-Dimer measurements, blood samples taken into sodium citrate tubes were centrifuged at 4000 rpm for 10 minutes, and their plasma was separated. D-Dimer levels were measured from the obtained plasma by immunoturbidimetric method, using an Innovance D-Dimer kit and Sysmex CS 2000i (Sysmex Corporation, Kobe, Japan) coagulation analyzer.

NT pro-BNP analysis was evaluated with the AQT 90 flex (Radiometer Medical Aps, Bronshoj, Denmark) device.

CRP levels were determined quantitatively by the immunonephelometric method using the appropriate kit in a Siemens BN II (Germany) device.

Troponin levels were determined by Abbott troponin ADV microparticle enzyme immunoassay on the Architect i2000SR Immunoassay Analyzer (Abbott Diagnostics, Chicago, Ill., USA).

The measurement of CK-MB activity was performed by the immune inhibition method using Abbott Architect c 8000 instruments (Abbott Diagnostics, Chicago, Ill., USA).

Procalcitonin (PCT) measurements were measured from serum by chemiluminescence method using Siemens Bayer Advia Centaur CP Immunoassay System (New York, USA) device and PCT kit (B.R.A.H.M.S. Diagnostica, Berlin, Germany).

### Echocardiography

Expert echocardiographers, blinded to the study plan, performed transthoracic echocardiography examinations using the Vivid 7® cardiac ultrasonography system (GE VingMed Ultrasound AS; Horten, Norway) with 2.5 to 5 MHz probes. Echocardiographic imaging was performed in left lateral and supine positions with parasternal long and short axes, and apical and subcostal windows were used to obtain Doppler tracings and 2D images. The measurements included LV ejection fraction (LVEF; modified Simpson method), pulmonary artery systolic pressure (PASP), tricuspid annular plane systolic excursion (TAPSE), pulmonary artery diameter (PAD), and right ventricle fractional area change (RV FAC) as per guidelines of the American Society of Echocardiography.^23^

### Statistical analysis

In all the statistical analyses, the SPSS version 14 (SPSS Inc., Chicago, IL, USA, institutional) software package was used. The two-sided p-value of < 0.05 was considered statistically significant. Number and percentage were used in expressing categorical variables; continuous variables were shown as mean±standard deviation (SD) or median and interquartile ranges (IQR), depending on their normality of distribution. The Kolmogorov-Smirnov test was used to determine the normality assumption of the data. In the comparison of the continuous variables, an independent sample t-test and a Mann-Whitney U test were used according to data normality. To compare the categorical data, an appropriate Chi-square test was used. In correlation analyses, a Pearson correlation test was preferred for variables that are normally distributed, and a Spearman correlation test was used for non-normally distributed variables. For the prediction of 6-month mortality and/or recurrent Cardiovascular-Related hospitalization using the receiver operator characteristic (ROC) curve analysis MedCalc (v12.7.8, personal registration), an optimal cut-off threshold was found for sST2 level, which was achieved by determining the area under the curve (AUC) with a 95% confidence interval. The best cut-off value for 6-month mortality and/or recurrent Cardiovascular-Related hospitalization was obtained by the calculation of the highest sum of sensitivity and specificity-1. Using the enter method, a logistic regression model with a single variable was constructed. Using the Pearson test for parametric data and the Spearman test for ordinal data, a univariate correlation was determined. A univariate analysis was performed for the primary endpoint in this study. In order to determine the independent predictors of 6-month mortality and/or recurrent Cardiovascular-Related hospitalization, the variables having significant association in the univariate analysis were entered in the multivariate logistic regression model using the backward stepwise method along with other potential confounders.

## Results

The mean age of 100 patients (39 males, 61 females) included in the study was 65.5±17.5 years. A total of 27 (27%) patients died, 6 of them (6%) during the index hospitalization, and 21 (21%) died during the 6-month follow-up. Nineteen patients had hospitalizations. Patients were classified into two groups: group 1, including patients (46) with composite outcome of 6-month mortality and/or recurrent cardiovascular-related hospitalization during follow-up, and group 2 patients (54) without composite outcome. In the first group, 26 patients were included because of 6-month mortality and 20 patients because of cardiovascular-related hospitalizations. The two groups were similar in terms of age, body mass index (BMI) during index hospitalization, gender distribution, hypertension (HT), diabetes mellitus (DM), smoking status, presence of cancer, and deep vein thrombosis. Syncope at admission was significantly more frequent in group 1. The PESI scores of the patients in group 1 were statistically significantly higher than those in group 2. (Table 1) 7 % of the patients were classified as having a very low risk, 9 % as having a low risk, 11 % as having a medium risk, 14 % as having a high risk, and 59 % as having a very high risk. Analyzing the distribution between groups revealed that 37 patients in the group with mortality and readmission had a very high risk, 4 had a high risk, 1 had a medium risk, 3 had a low risk, and 1 had a very low risk. In the group without mortality and readmission, six patients had a very low risk, six had a low risk, ten had an intermediate risk, ten had a high risk, and twelve had a high risk. In the group of patients with readmission and mortality, 35 of 41 high and very-high-risk patients received thrombolytic therapy. 17 of 22 very high and high-risk patients in the group received thrombolytic therapy without recurrent hospitalization or mortality. In both categories, all patients at extremely high risk received thrombolytic therapy, and there were no fatalities due to thrombolytic therapy-related complications. Six patients developed chronic thromboembolic pulmonary hypertension during the six-month follow-up. Upon evaluation of sST2 levels, the group that developed CTEPH had significantly higher sST2 levels. (172.7±75.6 vs 88.8±75.9 ng/mL; p= 0.041). In our study, the in-hospital mortality rate was 12%. The mortality rate at 6-month follow-up was 26%

**Table 1:** Baseline characteristics, laboratory significance, echocardiographic parameters

Characteristics		6-month mortality and/or recurrent cardiovascular hospitalization (n:46)	No 6-month mortality and/or recurrent cardiovascular hospitalization (n:54)	P
Age, years		68.9±15.7	62.6±18.6	0.076
Sex		18 (%39)	21 (%40)	1.000
BMI, kg/m^2^		27.6±4.0	28.3±3.2	0.743
Hypertension		23 (%50)	24 (%45)	0.639
Diabetes mellitus		12 (%26)	7 (%13)	0.172
Hyperlipidemia		17 (%37)	13 (%25)	0.267
Smoker		11 (%24)	10 (%19)	0.741
Cancer		14 (%30)	7 (%13)	0.065
DVT		18 (%39)	28 (%53)	0.173
Syncope		15 (%33)	6 (%11)	0.019
Chest pain		20 (%44)	16 (%30)	0.245
S1Q3T3		6 (%13)	9 (%17)	0.792
PESI score		181±71	127±59	<0.001
**Laboratory significance**				
	CRP, mg/L	73 (21.6-134)	41 (16.8-88)	0.040
	PRC, ng/mL	0.11 (0.05-0.55)	0.07 (0.02-0.14)	0.012
	NT-pro BNP, pg/mL	992 (397-2855)	360 (233-1495)	0.009
	D-dimer, ng/mL	4.7 (2.0-12.6)	4.1 (1.7-11.6)	0.664
	CK-MB, ng/mL	3.5 (1.5-5.8)	1.7 (0.9-4.3)	0.040
	Troponin I, ng/mL	0.09 (0.01-0.95)	0.04 (0.02-0.47)	0.624
	sST-2, ng/ml	138.6 (56.7-236.8)	38 (26.3-75.4)	<0.001
**Echocardiographic parameters**				
	TAPSE, mm	13.3±3.5	16±3.8	0.001
	sPAB, mmHg	40±9.9	37±9.9	0.150
	RV EF, %	36.6±7.5	38.6±7.7	0.206
	PA diamater, mm	29.2±4.6	27.2±4.2	0.026
	RV diamater, mm	38.2±6.3	35.1±6.4	0.001

IBMI: body mass index; DVT: deep vein thrombosis; S1Q3T3:D1 S wave, D3 Q wave, D3 T wave; PESI: pulmonary embolism severity index; CRP: C-reactive protein; PRC: procalcitonin; NT-PRO BNP: n-terminal prohormone brain natriuretic peptide; CK-MB: creatine kinase–MB; TAPSE: tricuspid annular plane systolic excursion; sPAB: systolic pulmonary artery pressure; EF: ejection fraction; RV: right ventricle; PA: pulmonary artery.

In the laboratory tests, CRP, procalcitonin, pro-BNP, CK-MB, and sST2 levels were higher in group 1 compared to group 2 respectively (Table 1).

In echocardiographic evaluation, right ventricular ejection fraction (EF), systolic pulmonary artery pressure, and left ventricular EF were similar in both groups. TAPSE was significantly lower in group 1 than in group 2. Right ventricular diameter and pulmonary artery diameter were significantly higher in group 1 than in group 2, respectively (Table 1).

While sST2 levels were positively correlated with age, PESI score, systolic pulmonary artery pressure, pulmonary artery diameter, troponin, and CK-MB, it was negatively correlated with TAPSE (Table 2).

**Table 2:** Correlation Coefficients for soluble ST-2 levels

**Age**	0.212	0.035
**PESI**	0.459	<0.001
**TAPSE**	-0.408	<0.001
**sPAB**	0.280	0.005
**PA diamater**	0.258	0.010
**CK-MB**	0.316	0.001
**Troponin I**	0.276	0.006

PESI: pulmonary embolism severity index; TAPSE: tricuspid annular plane systolic excursion; sPAB: systolic pulmonary artery pressure; PA: pulmonary artery; CK-MB: creatine kinase–MB.

The best cut-off threshold for sST2 levels in the prediction of 6-month mortality and/or recurrent Cardiovascular-Related hospitalizations was found to be >89.9 ng/mL with a specificity of 90.6% and a sensitivity of 65.2%, according to the receiver operating characteristic curve (area under the curve = 0.798; 95% CI, 0.705–0.891; *p* <0.0001). (Central Illustration)

Figure 1 also depicts the comparison between pro-BNP, a direct marker of ventricular overload, and sST2 (p=0.009).

**Figure 1:** Receiver operator characteristic (ROC) Curve of soluble ST-2 levels and NT-pro BNP levels to predict 6-month mortality and/or recurrent cardiovascular hospitalization.

Figure 2 depicts the comparison between PESI, which determines the risk of mortality and severity of complications and sST2 (p=0.175).

**Figure 2:** Receiver operator characteristic (ROC) Curve of soluble ST-2 levels and PESI score to predict 6-month mortality and/or recurrent cardiovascular hospitalization.

After adjusting for confounding factors that were either statistically significant in the univariate analysis or for the variables correlated with the sST2 levels, the sST2 level and CRP continued to be significant predictors of a composite outcome of 6-month mortality and/or recurrent cardiovascular-related hospitalizations in the multiple logistic regression model via backward stepwise method (Table 3).

**Table 3:** Univariate and multivariate logistic regression analysis representing the independent predictors of 6-month mortality and/or recurrent cardiovascular hospitalization

Variables		Univariate		Multivariate	
		OR (95% CI)	p	OR (95% CI)	p
sST-2		1.021 (1.012-1.030)	<0.001	1.019 (1.009-1.028)	<0.001
CRP		1.009 (1.002-1.017)	0.014	1.010 (1.001-1.021)	0.046
NT-pro BNP, pg/ml		2.838 (1.281-6.286)	0.010		
Procalcitonin		1.008 (0.914-1.111)	0.878		
CK-MB		1.140 (0.993-1.309)	0.064		
PESI		1.012 (1.006-1.011)	<0.001		
Syncope		3.790 (1.327-10.829)	0.013		
TAPSE		0.831 (0.744-0.928)	0.001		
PA diameter, mm		1.108 (1.010-1.215)	0.029		
RV diameter, mm		1.079 (1.011-1.152)	0.021		
**Correlated value with sST2**					
	Age, years	1.022 (0.997-1.046)	0.080		
	TROPONIN I,ng/ml	1.216 (0.786-1.881)	0.380		

CRP: C reactive protein; NT-PRO BNP: n-terminal prohormone brain natriuretic peptide; CK-MB: creatine kinase–MB; PESI: pulmonary embolism severity index; TAPSE: tricuspid annular plane systolic excursion.

## Discussion

In this study, sST2 level was an independent predictor of composite outcome of 6-month mortality and/or recurrent cardiovascular-related hospitalization in acute PE patients. In addition, serum CRP levels were also found to be an independent predictor of the composite outcome.

IL-33 is secreted from cells upon cell damage and necrosis, cells under mechanical stress, and in the inflammatory process. IL-33 binds ST2L, yielding inflammatory gene transcription and creating an inflammatory response with the release of inflammatory chemokines and cytokines. The inflammatory response results in apoptosis and fibrosis. Soluble ST2, on the other hand, binds with IL-33 and suppresses this inflammatory response, thus generating a defense mechanism to suppress apoptosis and fibrosis.^24-28^ The main source of circulating sST2 in healthy individuals and patients with different diseases is not fully elucidated. Experimental data suggested that mechanical stress could induce the expression of both IL-33 and sST2 in cardiomyocytes and fibroblasts.^29^ It was shown that sST2 levels were linked to mortality and prognosis in acute and chronic HF in relation to mechanical stress and inflammatory processes within the left ventricle.^19-21,30^ Besides, sST2 levels in PH patients were shown to be closely related to hemodynamic parameters, especially right ventricular dysfunction, and to be a marker of mortality.^22^ Similarly, in our study, sST2 levels were found to be an independent predictor of composite outcomes in PE patients. Obstruction due to a thrombus in PE generates dead spaces in the lung yields an inflammatory process and hypoxia. The inflammatory process and hypoxia cause vasoconstriction in the pulmonary bed, resulting in increased pulmonary vascular resistance (PVR) and pulmonary artery pressure, which can increase filling pressure in the right ventricle and hence increase sST2 levels. Therefore, sST2 may be a good biomarker to reflect right ventricular loading. Of note, right ventricular loading is a marker of mortality in PE patients.^4-6^ Hence, sST2 levels can be used as a surrogate for poor outcomes. On the contrary, during the inflammatory process related to acute PE, sST2 may bind IL-33 and play a role in inflammation suppression, fibrosis, and remodeling prevention. There was supporting evidence with regard to vascular endothelium-derived sST2 in the literature.^31^

It has long been recognized that inflammation plays a role in the pathogenesis of arterial and venous thrombosis by activating coagulation cascades through thrombin generation and fibrin deposition. Arterial and venous thrombosis is influenced by inflammatory mediators, specifically CRP, IL-6, IL-8, and tumor necrosis factor.^32,33^ In limited animal investigations, it has been demonstrated that inflammation following PE contributes to RV injury, dysfunction, and cardiac inflammation.^34^ PE can induce vascular inflammatory reactions via thromboembolism of the pulmonary artery, as well as pulmonary parenchymal inflammation via pulmonary infarction mimicking pneumonia. Thromboembolism of the pulmonary artery can hasten RV dysfunction via cardiac inflammation that contributes to myocyte injury. C-reactive protein can be used to assess the risk and prognosis of myocardial ischemia and ischemic stroke based on the pathophysiology of arterial thrombosis.^33^ It has also been shown in previous studies that high CRP, another inflammatory marker, is also associated with poor prognosis in PE patients.^32,33^ In our study, CRP levels were associated with 6-month mortality and/or recurrent cardiovascular-related hospitalizations.

Pro-BNP, which rises in response to right ventricular strain; CK-MB, which indicates right ventricular injury; and procalcitonin, which rises as a result of the inflammatory process in PE, were found to vary between the groups. Similarly, echocardiographic data such as TAPSE, pulmonary artery diameter, and RV diameter, which are indicators of right ventricular echocardiographic function, were discovered to vary between the groups. Echocardiographic data and laboratory data were found to be predictors of mortality and readmissions in univariate analysis. Unlike the literature, troponin values were found to be similar in the two groups in our study population. Since most of the patients included in the study were high-risk patients, troponin values were above the normal range but were similar between the groups. Only sST2 and CRP were discovered to be independent predictors in multivariate analysis. This may be because our facility is a tertiary center, and the majority of our patients are high-risk patients. In addition, both mortality and mortality associated with recurrent hospitalizations may have contributed to the independence of sST2 and CRP as predictors.

### Limitations

The present study has some limitations. One of the limitations of our study is its single-centered nature, which prevents findings from being generalized to the overall population of patients with acute PE with varying degrees of severity. Besides, the sample size is not large enough to draw definitive conclusions. Of note, other inflammatory markers, such as IL-1, IL-6, and TNF-alpha, which could add to sST2, were not measured.

## Conclusion

sST2 level looks like a biomarker that can be used to predict a 6-month composite outcome in high-risk acute PE patients, confirming its close relationship with right ventricular loading and its role in the inflammatory process.
